# User Experience Regarding Digital Primary Health Care in Santarém, Amazon: Evaluation of Patient Satisfaction and Doctor’s Feedback

**DOI:** 10.2196/39034

**Published:** 2023-01-11

**Authors:** Kaio Jia Bin, Patrícia Gabriela Santana Alves, Raquel Costa, Paula Cruz Eiras, Luciano Nader de Araujo, Antonio José Rodrigues Pereira, Carlos Carvalho, Ana Maria Malik

**Affiliations:** 1 Hospital das Clínicas Faculdade de Medicina Universidade de São Paulo São Paulo Brazil; 2 Faculdade de Medicina Universidade de São Paulo São Paulo Brazil; 3 Fundação Getúlio Vargas Escola de Administração de Empresas de São Paulo São Paulo Brazil

**Keywords:** telemedicine, primary health care, user’s experience, Amazon, digital health, pilot, patient, pilot model, pandemic, medical care, assist, urban, community, Brazil, technology, consultation, physician, survey

## Abstract

**Background:**

With the arrival of the pandemic, telemedicine has been widely used to provide medical care and can be used to assist patients in regions far from urban centers that are difficult to access, such as riverside communities in the Brazilian Amazon region. A telemedicine project connecting São Paulo, a mega-metropolis, to Paysandú, a riverside district in the Amazon, was built to serve the local population where access to the nearest medical care is 6 hours away by speedboat.

**Objective:**

This study aims to assess the feedback from patients and doctors regarding the use of telemedicine in outpatient care at Paysandú, a riverside district in the Amazon.

**Methods:**

This is a single-center study following the guidelines “Evaluating digital health products” from Public Health England, with local adaptations for the project and the Brazilian reality, that was conducted between São Paulo and Santarém in Brazil. A survey was carried out with patients who were treated by a doctor in the city of São Paulo, about 2500 km from the local basic health unit, between September 27 to December 15, 2021. At the end of each teleconsultation, the attending physician answered an administrative survey form, and the patient answered a satisfaction survey.

**Results:**

A total of 111 patients completed the satisfaction survey from a total of 220 consultations carried out during the period (95% CI margin error 0.22%). According to the survey, more than 95% of patients were satisfied with the service, 87.4% (n=97) had previous experience with videoconferencing, and 76.6% (n=85) reported that their demand was fully solved. Additionally, according to the hired doctor’s feedback, the average duration of the consultations was between 15 and 20 minutes. Of the 220 teleconsultations performed, 90.9% (n=200) of the demands were solved with support from the local health team, and 99.1% (n=218) of the appointments had a problem with audio or video.

**Conclusions:**

This teleconsultation project between São Paulo and Paysandú showed that it is possible to offer medical care from more developed locations to communities far from urban centers, as is the case with Paysandú District. Beyond the feasibility of the infrastructure, acceptance and satisfaction among patients were high. This health care supply model has proven to be functional and should be expanded nationally or perhaps internationally to regions lacking medical assistance. Escalation of the project does not seem too difficult once infrastructure issues are solved.

## Introduction

Until 2020, the last alert for a pandemic had been declared by the World Health Organization more than 10 years earlier, referring to the global spread of the H1N1 virus in 2009 [1]. In 2020, the level of contamination by a new type of coronavirus with great infective potential became global, leading to a new World Health Organization statement warning about the COVID-19 pandemic on March 11 [2].

In early 2020, the Brazilian Ministry of Health had already declared an Emergency in Public Health of National Importance [3], leading to a series of responses from the state and local health systems to fight the disease. These responses modified daily activities for the entire population. With the need for social distancing, different levels of care sought alternatives to maintain regular activities for their populations, encouraging the incorporation of new technologies into services and boosting existing initiatives. In this context, services mediated by technology gained prominence, including telehealth or telemedicine actions to support diagnosis, therapy, and health education, especially where distance is a critical factor [4].

Telemedicine and telehealth actions have already been incorporated by other health systems in the world as is the case of Canada, which, given the geography and often adverse climate, was one of the first countries to use IT tools, especially starting in the 1990s, to provide medical care and promote distance education [5]. In the United Kingdom, the National Health Service has also adopted telemedicine since the 1990s and considers it a method that combines the use of computers and telecommunication technology with medical expertise to send and receive information that helps a medical diagnosis and that assists services in caring for people who are far away [6]. In Brazil, the use of telemedicine was controversial until Ordinance N.467 of March 20, 2020, edited by the Ministry of Health, that allowed the use of telemedicine actions covering health care and teleconsultations during the pandemic due to the need to reduce the circulation of people exposed to the new virus [7], especially in health services.

Despite telemedicine being considered a recent advancement in Brazil when compared to its diffusion in high-income countries such as England, Canada, and the United States, it is an opportunity to increase equity in the access and delivery of medical care, regardless of the geographic location and socioeconomic status of users of the health system [8]. Several Brazilian national and local government initiatives are currently underway. One of them is the Telessaude Brasil Program that started in 2019 with the aim of improving and expanding the health services network, mainly strengthening the communication of primary care with other levels of health care [9].

Another Brazilian government initiative is the University Telemedicine Network Project (Rede Universitária de Telemedicina) by the Ministry of Health and the Ministry of Technology and Innovation, which aims to support the telemedicine infrastructure in universities and teaching hospitals countrywide to promote greater collaboration, communication, health care, and teaching [10].

Within the current pandemic context, the need to strengthen primary health care was explicit; in addition to the scope of prevention, promotion, and health education innate to the different levels of care, primary care also proved to be an important resource in combating COVID-19 [11].

To preserve primary health care’s routine activities, it is necessary to readjust and to incorporate new procedures, including the use of information and communication technologies to carry out teleconsultations [11].

Teleheath enables the system to avoid treatment discontinuity or the worsening of patients with chronic conditions, in addition to meeting lower risk demands, which do not require face-to-face interaction, for example, the renewal of prescriptions for continuous use [11].

A qualitative systematic review published in 2020, which analyzed the results of 43 studies in different countries on the perception of health professionals working with telemedicine programs in primary health care, showed that the innovation has improved the relationship between practitioners and their patients as well as enabled the delivery of care in regions difficult to access. A counterpoint described in the same study is the difficulty of maintaining this type of care when it depends on the availability of individual equipment—cell phones, tablets, computers—by the target population [12].

In view of this evidence, a solution that promotes remote primary health care delivery through teleconsultations that is not conditional on the availability of technological resources for personal use by each user of the system is needed, allowing for interaction between people physically located kilometers away and providing quality care capable of meeting the demands of a region lacking health professionals [13].

Social distancing as a measure of sanitary control leveraged the development of telemedicine, turning it into an important health care tool for individuals who, historically, have little access to health services [14].

Given the incorporation of new technologies and new modes of care delivery to users of the health system, it is important to adopt methodologies that assess new work processes. It should not only consider the results and impacts but also enable measurement of satisfaction with the new modalities of care among users. Issues such as access to the service and doctor-patient relationship are fundamental aspects of the health care model when it comes to user satisfaction [15].

In Brazil, a continental country, some cities are among the most developed in the world, like the city of São Paulo. On the other hand, some of them, with a very low Human Development Index, can only be accessed by boat in the middle of the Amazon region like the district of Paysandú. For these hard-to-reach small towns, even access to primary care provided by a primary care physician is often scarce. In Society 5.0, where humans are supposed to be the center and technology the servant, geographical barriers should be broken using telemedicine.

Based on the Scrum methodology already used previously [16], a telemedicine project was carried out between the city of São Paulo and Paysandú, a riverside district of the city of Santarém in the Amazon region of Brazil, between September 27 and December 15, 2021. At the end of each consultation, a survey of patient satisfaction and physician impressions were collected over 220 performed teleconsultations.

This study aims to assess the feedback from patients and doctors regarding the use of telemedicine in outpatient care at Paysandú, a riverside district in the Amazon.

## Methods

### Study Type

A single-center study following the guidelines “Evaluating digital health products” from Public Health England [17], with local adaptations for the project and the Brazilian reality, was conducted between São Paulo and Santarém in Brazil.

A descriptive study of the “analysis of routinely collected data” [18] using administrative and “user feedback study” [19] data collected by a satisfaction survey was conducted by the management team.

### Ethics Approval

This user experience study was approved by the ethics committee of Hospital das Clínicas, Faculdade de Medicina, Universidade de São Paul Comissão de Ética para Análise de Projetos de Pesquisa) without the need for ethical analysis by the research ethics committee system (Comitês de Ética em Pesquisa/Conep). According to the guidelines of Brazilian National Health Council resolution No. 466/12 and No. 510/16, studies that only have the objective of assessing a service for the purpose of its improvement or implementation, which do not aim to obtain generalizable knowledge but only data that can be used by that service for its improvement, do not require ethical analysis by the research ethics committee system (Comitês de Ética em Pesquisa/Conep), as it does not have direct patient involvement or handle personal data [20,21].

In this study, a satisfaction survey was carried out among patients, and an opinion survey was given to physicians. All responses were collected anonymously, without identifying or sensitive data.

All the data collected were used to evaluate the performance of the pilot project carried out between September and December 2021 to identify possible points for improvement, such as internet connectivity and patient satisfaction.

### Main Research Question

What is the satisfaction of patients regarding teleconsultation between São Paulo and Paysandú?

### Study Setting

From September 27 to December 15, 2021, a study was carried out linking a basic health unit in the city of Santarém and a university hospital in the city of São Paulo, separated by about 2500 km. The basic health unit is located in Paysandú District, a riverside city in the city of Santarem, state of Pará, Brazil. The local team consists of 1 nurse and 2 nursing technicians.

For remote medical care, Hospital das Clínicas, Faculdade de Medicina, Universidade de São Paulo, a tertiary university hospital, hired a primary care physician for 20 hours per week (5 hours per day, 4 days per week on Monday, Tuesday, Wednesday, and Friday afternoons), totaling 39 days with 195 hours scheduled for appointments and 220 hours performing teleconsultations (Table 1).

Disregarding holidays, local vaccination campaigns, and justified absences of the hired doctor when there were no remote appointments during the project, there were 220 performed consultations (Table 1).

**Table 1:** Total planned days, total planned hours, and total performed consultation per week during the project.

Week	Total planned days (N=39), n	Total planned hours (N=195), n	Performed consultation (N=220), n
Week 1	3	15	10
Week 2	4	20	0
Week 3	2	10	8
Week 4	4	20	32
Week 5	4	20	22
Week 6	3	15	10
Week 7	4	20	20
Week 8	3	15	27
Week 9	3	15	19
Week 10	3	15	28
Week 11	3	15	21
Week 12	3	15	23

### Telemedicine Performance

To assess the performance of telemedicine, at the end of each consultation, the doctor fills out a multiple-choice survey based on their personal opinion through a Google Form on the administrative aspects of care.

To simplify the administrative analysis, the consultation performed was classified according to the following criteria by the doctor:

- Investigation: presents acute complaints to be analyzed; follow up with tests for diagnostic confirmation and re-evaluation
- Orientation: guidelines regarding procedures, contraceptive methods, and prescription renewal

### Patient’s Satisfaction Survey

At the end of each medical consultation, a health agent asked whether the patient was interested in participating in a satisfaction survey anonymously. If the patient agreed to participate in the survey, a self-explanatory printed satisfaction survey in Brazilian Portuguese with multiple-choice questions using a Likert scale was given to the patients, with the following response options:

- I totally agree
- I partially agree
- Indifferent
- I partially disagree
- I totally disagree

The questions used in the satisfaction survey (translated from Portuguese):

1. Waiting time to get an appointment with the doctor was satisfactory.
2. Duration of medical care was satisfactory.
3. Doctor was respectful, kind, and considerate during the consultation.
4. Doctor’s explanation of care/treatment plan was clear and understandable.
5. You feel confident with the care/treatment plan recommended by the doctor.
6. You feel that your privacy was respected throughout the service.
7. Your demand was appropriately met by the doctor.
8. You are already familiar with video calls via cell phone or computer.
9. Local team provided you with the necessary guidance before remote medical care.
10. Local team provided you with the support needed during the video call.
11. Audio quality during the video call was satisfactory.
12. Image quality during video call was satisfactory.
13. You feel comfortable receiving medical care by video call.
14. Remote care provided by the doctor was as good as face-to-face care.
15. Remote medical care has made it easier for me to see a health care provider.

### Study Design

The data from Google Forms were extracted to Excel version 2010 (Microsoft Corporation), where they were analyzed.

## Results

From the 220 performed appointments, 160 were previously scheduled and 60 were scheduled on the same day of the consultation (Table 2).

Of the 220 performed teleconsultations, 70.9% (n=156) were a visit with a clinical investigation, 90.1% (n=200) took between 15 and 20 minutes, 80.9% (n=178) were first visits, 70% (n=154) received medical discharge, and only 2.7% (n=6) needed local specialist medical care support; none required local generalist medical care (Table 3).

Regarding the question “Was it possible to solve the patient's demands?” the hired doctor answered that in 90.1% (n=200) of visits, it was possible to solve the patient’s demands with the support of the local team (Table 4).

About “connectivity issues?”, the doctor answered that, in 75.5% (n=166) of the visits, there were issues with audio and video; only 2 visits were performed without any connectivity issue related to audio or video (Table 5).

The satisfaction survey received a total of 111 answers of the 220 total performed consultations; at a 95% CI, the margin of error was 0.22% (Table 6).

About the remote medical care, according to satisfaction survey responses, more than 96% of patients agreed that:

- Waiting time to get an appointment with the doctor was satisfactory;
- Duration of medical visit was satisfactory;
- Doctor was respectful, kind, and attentive during the consultation;
- Doctor’s explanation of care/treatment plan was clear and understandable;
- Remote care provided by the doctor was as good as face-to-face care;
- Remote medical care has made it easier for you to see a health care provider; and
- Local team provided you with the necessary guidance before remote medical care.

About the connectivity of the remote medical care, 87.4% (n=97) of patients agreed that they were already familiar with using video calls via cell phone or computer, and more than 95% agreed that:

- Audio quality during the video call was satisfactory,
- Image quality during the video call was satisfactory, and
- Local team provided the support needed during the video call.

Regarding the patient, more than 97% of them agreed that they:

- Felt confident with the care/treatment plan recommended by the doctor,
- Felt that privacy was respected throughout the consultation, and
- Felt comfortable receiving medical care by video calling.

A total of 76.6% (n=85) of patients agreed that their demands were appropriately met by the doctor. Table 7 presents the detailed distribution of answers by question regarding satisfaction survey.

**Table 2:** Total service performance by type of attendance.

When the appointment was scheduled	Consultation (N=220), n (%)
Previous scheduled	160 (72.7)
Scheduled at same day	60 (27.3)

**Table 3:** Administrative information collected after medical teleconsultation.

			Consultation (N=220), n (%)
**Was it an investigation or an orientation?**			
	Investigation	156 (70.9)	
	Orientation	64 (29.1)	
**What was the duration of the consultation? (minutes)**			
	10-15	6 (2.7)	
	15-20	200 (90.9)	
	>20	14 (6.4)	
**Was it first visit or return?**			
	First visit	178 (80.9)	
	Return visit	42 (19.1)	
**Did it require a return visit or led to medical discharge?**			
	Medical discharge	154 (70.0)	
	Return for re-evaluation	30 (13.6)	
	Return with complementary exams	36 (16.4)	
**Did it require local medical care?**			
	Forwarding to local specialist	6 (2.7)	
	Forwarding to local generalist	0 (0.0)	
	None	214 (97.3)	

**Table 4:** Was it possible to meet the patient’s demands?

Was it possible to meet the patient’s demands?	Consultation (N=220), n (%)
Yes, without local team support	6 (2.7)
Yes, with local team support	200 (90.9)
Partially, with local team support	14 (6.4)
No, needed an external medical care	0 (0.0)

**Table 5:** Were there any connectivity issues?

Were there any connectivity issues?	Consultations (N=220), n (%)
Problems with audio and video	166 (75.5)
Problems with audio	44 (20.0)
Problems with video	8 (3.6)
None	2 (0.9)

**Table 6:** Participation in satisfaction survey and margin of error.

Participation in satisfaction survey	Value
Total performed consultation, n	220
Total participation in satisfaction survey, n	111
CI (%)	95.0
Margin of error (%)	0.22

**Table 7:** Detailed distribution of answers (N=111) by question of the satisfaction survey.

	I totally agree, n	I partially agree, n	Indifferent, n	I partially disagree, n	I totally disagree, n	No reply, n	Agree (%)	Disagree (%)
Waiting time to get an appointment with the doctor was satisfactory	74	34	0	1	1	1	97	2
Duration of medical care was satisfactory	87	23	0	0	0	1	99	0
Doctor was respectful, kind, and attentive during the consultation	89	19	0	1	0	2	97	1
Doctor’s explanation of care/treatment plan was clear and understandable	87	22	0	0	1	1	98	1
Remote care provided by the doctor was as good as face-to-face care	85	22	2	1	0	1	96	1
Remote medical care has made it easier for you to see a health care provider	83	24	0	1	0	3	96	1
Local team provided you with the necessary guidance before remote medical care	84	23	1	1	1	1	96	2
You are already familiar with using video calls via cell phone or computer	70	27	2	2	2	8	87	4
Audio quality during the video call was satisfactory	78	29	0	0	1	3	96	1
Image quality during video call was satisfactory	84	21	2	0	3	1	95	3
Local team provided you with the support needed during the video call	89	20	0	1	0	1	98	1
You feel confident with the care/treatment plan recommended by the doctor	83	27	0	0	0	1	99	0
You feel that your privacy was respected throughout the consultation	90	18	0	0	1	2	97	1
You feel comfortable receiving medical care by video calling	89	20	1	1	0	0	98	1
Your demand was appropriately met by the doctor	76	9	0	0	0	26	77	0

## Discussion

### Principal Findings

A pilot project of telemedicine was built to connect a metropolitan city, rich in human resources and infrastructure, to a riverside city in the middle of the Amazon region, where the only possible physical access is by boat and the internet is provided by satellite. Before this pilot project, the local population of the district Paysandú received one medical visit a month from the city of Santarém, with 30 appointments scheduled for 1 day. When there is an unscheduled spontaneous demand, a 6-hour boat ride downtown is needed.

The pilot project provided the local population with one web-based physician 4 times per week; during the 12-week period, 220 consultations were offered (an average of 73 consultations every 4 weeks). Considering the infrastructure connectivity shortcomings, there was no need for the doctor of the city of Santarém to travel to the district of Paysandú for local consultations. To reduce subjective bias, only one clinical physician was hired to evaluate the conditions provided for telemedicine, like other studies involving technologies [22].

As described by Combi et al [23], implementing telemedicine in low-income countries has more challenges than in high-income countries. So, there are several relevant findings from the feedback from the hired clinical physician.

First, the web-based doctor did not need the support from a local doctor, which means that a permanent telemedicine program is possible in this region. Second, only 0.9% (n=2) of the teleconsultations had no connectivity issues, a recurring problem in remote regions such as Amazonas [23]. Even with signal problems, the presence of the doctor at a distance is useful for the local population, as will be discussed below. Third, 90.1% (n=200) of teleconsultations needed a local support team and relevant information regarding setting up a health care unit in remote regions, requiring the presence of a local health agent.

In addition to being effective, the telemedicine service also needs to be well accepted by patients [24,25], even with connection problems in more than 99% of teleconsultations. More than 95% of the survey participants were satisfied with the received health care, especially in relation with the doctor, the infrastructure (even with the existing connectivity problems), and the support of the local team. This percentage was probably directly influenced by the high percentage of patients who were familiar with the use of telemedicine [23]. However, it is noteworthy that 76.6% (n=85) of patients responded that they had their needs answered by the doctor during the teleconsultation (Table 7) using only a videoconference camera, which indicates that telemedicine helps substantially, but it still does not replace face-to-face care in full, and barriers still need to be overcome [26].

### Limitations

To avoid bias from different doctors in the performance of the consultations, all appointments were performed by a single hired doctor, which had the limitation of medical impressions being from a single source.

Additionally, because there was only one doctor in the study, the high rate of satisfaction may be due to the role played by the hired doctor; on the other hand, the high rate of satisfaction also suggests that a good doctor-patient relationship increases patient satisfaction even with medical care at a distance and scarce resources.

No evaluation was made comparing this visit with the previous monthly medical care visit, so it is not possible to assess whether telemedicine was better accepted in relation to previous medical care.

As this was a user experience study based on satisfaction and opinion, no clinical data were collected or evaluated; so, there is no way to assess the clinical effectiveness of telemedicine, only its acceptance by the involved users.

### Conclusion

This teleconsultation project between São Paulo and Paysandú showed that it is possible to offer medical care from more developed locations to communities far from urban centers.

Beyond the feasibility of the infrastructure, acceptance and satisfaction among patients was high. This remote health care model proves to be functional and should be expanded nationally or perhaps internationally to regions lacking medical care. Escalation of the project does not seem too difficult once infrastructure issues are solved.

Having the possibility of offering medical care with professionals at a distance, it is possible to design a future where members of a small community can go to urban centers in search of medical education and, after completing their graduation, serve their home population through telemedicine.

In addition to the possibility of using remote monitoring devices, such as smart watches [27], the potential for improving the quality of health care for populations in need of medical care is promising.
